# Mastering your fellowship

**DOI:** 10.4102/safp.v63i1.5319

**Published:** 2021-05-26

**Authors:** Mergan Naidoo, Klaus B. von Pressentin, Tasleem Ras, Hannes Steinberg

**Affiliations:** 1 Department of Family Medicine, College of Health Sciences, University of KwaZulu-Natal, Durban, South Africa; 2 Division of Family Medicine, Faculty of Health Sciences, University of Cape Town, Cape Town, South Africa; 3 Department of Family Medicine, University of the Free State, Bloemfontein, South Africa

**Keywords:** family physicians of South Africa, FCFP (SA) examination, family medicine registrars

## Abstract

The series, ‘Mastering your Fellowship’, provides examples of the question format encountered in the written and clinical examinations, Part A of the Family Physicians of South Africa (FCFP SA) examination. The series is aimed at helping Family Medicine registrars prepare for this examination. Model answers are available online.

## Introduction

This section in the *South African Family Practice* journal is aimed at helping registrars prepare for Part A of the Fellowship of the College of Family Physicians (FCFP SA) examination and will provide examples of the question formats encountered in the written examination: multiple choice question (MCQ) in the form of single best answer (SBA – Type A) and/or extended matching question (EMQ – Type R); short answer question (SAQ), questions based on the critical reading of a journal (evidence-based medicine) and an example of an objectively structured clinical examination (OSCE) question. Each of these question types are presented based on the College of Family Physicians blueprint and the key learning outcomes of the FCFP (SA) programme. The MCQs will be based on 10 clinical domains of family medicine, the modified essay questions (MEQs) will be aligned with the five national unit standards and the critical reading section will include evidence-based medicine and primary care research methods.

This month’s edition is based on unit standard 1 (critically reviewing new evidence and applying the evidence in practice, principles of self-care and leading a clinical governance team) and unit standard 2 (evaluate and manage a patient according to the bio-psycho-social approach). The domain covered in this edition is Mental Health. We suggest that you attempt answering the questions (by yourself or with peers/supervisors) before finding the model answers online: http://www.safpj.co.za/.

Please visit the Colleges of Medicine website for guidelines on the Fellowship examination: https://www.cmsa.co.za/view_exam.aspx?QualificationID=9.

We are keen to hear about how this series is assisting registrars and their supervisors in preparing for the FCFP (SA) examination. Please email us your feedback and suggestions.

### Multiple choice question: Single best answer

A registrar reports feeling emotionally exhausted, irritable, detached, negative to his work environment and having a low sense of accomplishment. He believes that the long working hours, high patient and academic load, limited resources, lack of support from seniors at work and the conflictual home environment have all contributed to his current mental state. What is the most appropriate next step?

- Advocate resilience training
- Ask him to reconsider his current employment
- Facilitate self-awareness and workload management
- Prescribe medication and refer for psychotherapy
- Refer him to the facility employee assistance programme

*Answer*: (c)

The scenario painted above is fairly common in the local context and often leads to all sorts of threats to personal and professional functioning. The symptoms described arecharacteristic of burnout and are a response to prolonged work stress because of workload pressures, resource constraints, lack of recognition of the contribution being made, low levels of support, poor decision-making and poor leadership. These pressures lead to emotional exhaustion, depersonalisation and a lack of accomplishment. The Maslach Burnout Inventory for medical personnel is a tool used to assess burnout scores and judge the severity of the burnout.

Risk factors of burnout are poorly functioning organisations. Qualities of a positive work setting include institutional functionality which has effective internal communication systems, high employee satisfaction, good support from management, being appreciated by patients and colleagues, harmonious work–family balance, good opportunities for personal development and effective leadership. Environments in which these positive attributes are lacking predispose doctors to developing burnout. Such environments typically require doctors to deal with an excessive workload, long working hours, excessive fatigue, strained emotional interactions, high cognitive demands from the complexity of the practice, restricted autonomy and the impact of structural and organisational processes. Poor communication and teamwork and poor social interactions in the workplace also contribute to heightened stress levels. Certain demographic factors such as younger age, female gender, conflictual marital status, long working hours and low levels of job satisfaction are also associated with higher levels of burnout. Burnout may often lead to conflict in the family environment and substance abuse. Certain disciplines of medicine may also predispose to increased levels of burnout.

Some of the preventative strategies to mitigate the effect of burnout amongst doctors are the following:

- modifying the institutional structure and work processes
- improving the acceptability between the institution and the individual doctor through tailored professional development programmes and improving support and mentorship
- initiatives to reduce stress and poor coping mechanisms through the facilitation of coping strategies and promoting healthy lifestyles.

Intervention once burnout is diagnosed is a much more complex process and is generally managed very poorly through the institutional employee assistance programme, especially in a dysfunctional environment. Building resilience and sending the individual back to the same environment is also counter-intuitive. The best solution requires a holistic approach and transformative leadership. Individual counselling should be focused on facilitating self-awareness, encouragement of fostering healthy supportive relationships and getting the individual to focus on what is working. Improving well-being and performance requires the promotion of healthy behaviours such as sleep hygiene, nutrition, energy management, self-compassion and the training in leadership, communication and teamwork through a process of coaching and mentoring.

Organisations need to acknowledge that burnout exists in the workplace and thus need to work on strategies to manage the workload, provide safe working hours and create a culture of support and understanding. Additional support processes include peer support psycho-social programmes, promoting a culture of caring for one another and ongoing reflection, feedback, monitoring and evaluation.

#### Further reading

- Zeijlemaker C, Moosa S. The prevalence of burnout amongst registrars in the School of Clinical Medicine at the University of the Witwatersrand, Johannesburg, South Africa. S Afr Med J. 2019;109(9):668–672. https://doi.org/10.7196/SAMJ.2019.v109i9.13667
- Kumar S, editor. Burnout and doctors: Prevalence, prevention and intervention. Healthcare. 2016;4(3):37. https://doi.org/10.3390/healthcare4030037

### Short answer question: The family physician’s role as care provider and capacity builder

You have recently joined the staff of a district hospital where you are the only family physician.

In the 8 months that you have been covering casualty, you observe a number of the same patients returning with psychosis despite having been admitted to the ward.

#### 1. Conduct a root cause analysis using a fishbone diagram. (9 marks)

Model answer:

- Fishbone diagram to be drawn
- Problem (the head): Frequent re-admissions of known mental health users with psychosis: recurrent readmissions of patients with schizophrenia and/or bipolar patients. (1 mark)
- Identify the key problem and identify at least four categories of possible causes leading to the key problem. (1 mark each for each bone coming off the fish spine that has an appropriate heading with the underlying factor stated, up to a total of 8). Categories could include the following: policies related; people related; process/procedure related; environment related)
- Deconstruct these categories into possible underlying issues that may be related to the key problem.

These could be:

- Processes and procedures/therapy-related (e.g. adequate support systems/reminders/treatment buddies, involvement of ward based outreach team (WBOT)/ non-governmental organisations (NGOs), side effects of drugs)
- Policy-related: Healthcare system-related (e.g. appointment system, up and down referral issues, poor communication, lack of community-based care; medication stock-outs) (some of these would also be correct if put under processes)
- People-related: Healthcare worker-related (e.g. lack of being person-centred care, lack of training and stigma) and/or patient-related (e.g. poor insight or understanding, disorganised behaviour and thoughts, added substance abuse and blunting from drugs)
- Environment: Contextually related (social determinants of health – poverty, transport, poor social support from family, topography, culture, poor education, lackof respite for care-givers).

#### 2. Now that you have completed the root cause analysis, you decide to intervene. Using the I-We-It model of transformative leadership, outline your approach to address some of the issues you identified in the fishbone analysis. For each category of the I-We-It model below, provide one response that specifically addresses one of the identified issues. (6 marks)

Model answer:

- I: Personal transformation. Candidate acknowledges and reflects on his or her own personal values regarding mental health patients. A candidate should demonstrate an awareness of his or her own personal values and attitudes with regard to mental healthcare users. Examples of such a reflective response might include the following: fear experienced during psychiatric encounters when patients are aggressive, personal or family experience with suicide/depression/psychiatric disease with possible transference, managing one’s own prejudice/stigma, and over-identification with patients. (2 marks)
- We: Answer should indicate how they will build relationships and enable team work to practically improve mental health care. To get full marks, the candidate should choose a specific issue in the fishbone analysis and state how they will use relationships or teamwork to address the issue. (2 marks)
- It: Transformation of the district health system (DHS). To get full 2 marks, the candidate should choose an identified issue from the fishbone and state how it can be addressed by improving strategies or processes in the district health system. For example, the strategies could include creating better communication between levels in the referral system to improve efficiency, improving primary health care workers’ knowledge at district level by engaging specialists with outreach to clinics. (2 marks)

#### 3. List five indicators that you can use to conduct monitoring and evaluation once you have implemented your interventions. (5 marks)

Model answer:

- List of current mental healthcare providers for psychiatric patients per facility
- Number of missed outpatient appointments
- Number of readmissions within one year
- Number of patients failing to pick up prescriptions
- Number of patients that fail to receive depot injections
- Stock audits of psychiatric medications (periodic)
- Number of acute presentations
- Number of patients accompanied by South African Police Service (SAPS), family members, or coming alone
- Number of service providers engaged on stigma
- Number of continuous professional development (CPDs) provided
- Number of providers trained on the *Mental Health C are Act*

[20 marks]

#### Further reading

- Mash B, editor. Handbook of family medicine.4th ed. Cape Town: Oxford University Press Southern Africa. 2017; p. 365–370, 375.

### Critical appraisal of quantitative research

Read the accompanying article carefully and then answer the following questions (total 40 marks). As far as possible, use your own words. Do not copy out chunks from the article. Be guided by the allocation of marks with respect to the length of your responses:

- Mashaba BL, Moodley SV, Ledibane NR. Screening for depression at the primary care level: Evidence for policy decision-making from a facility in Pretoria, South Africa. S Afr Fam Pract. 2021;63(1):e1–e7. https://doi.org/10.4102/safp.v63i1.5217

1. What research question did the authors attempt to answer in this study? Comment on whether this was a clearly focused question. (5 marks)
2. Considering the introduction section in this article, identify three sentences or phrases that best reflect the authors’ starting point, from which the rationale for the research is further explained and/or elaborated (more than one correct answer possible). (3 marks)
3. Comment critically on the sample used in this study and any source of selection bias. (5 marks)
4. Critically appraise how well the authors describe the validity and administration of the study instrument identified for use in this study. (6 marks)
5. Critically appraise the reported response rate of 99.5%. (3 marks)
6. Critically appraise the authors’ decision to use a cut-off score of 5 to interpret the patient health questionnaire-9 (PHQ-9) score. (3 marks)
7. ‘Employed participants had significantly lower odds (odds ratio [OR] = 0.48) of screening positive (*p* = 0.033) for depressive features, whilst the participants with significantly higher odds of being screened positive were those with co-morbidities (OR = 2.12; *p* = 0.029) and a history of stressful life events (OR = 3.21; *p* = 0.001)’. Define and explain what you understand by this statement? (5 marks)
8. Use the acronym READER (Relevance, Education, Applicability, Discrimination, Evaluation and Reaction) to analyse this article’s applicability to your own context (take home message). (10 marks)

(Total: 40 marks)

### Model answers

#### 1. What research question did the authors attempt to answer in this study? Comment on whether this was a clearly focused question. (5 marks)

The authors attempted to determine the number of patients who screened positive for depressive features in adult patients who attended a primary healthcare (PHC) facility in Pretoria, Tshwane, South Africa, during February 2018. They also sought to identify risk factors associated with the positive screening for depressive features in this patient group. They hoped that their study findings could provide sufficient evidence to assist decision-making at PHC level with respect to screening for depression.

The Patient group, Patient Problem or Population of interest, Intervention or Issue of Interest, Comparison intervention of interest, primary outcome of interest (PICO) framework is generally used to help frame or focus the research question. The framework may be tailored to the research question type (treatment, prevention, diagnosis, prognosis or aetiology) or study design (quantitative compared to qualitative).

Using the PICO framework for this study, the population of interest (P) would be adult patients attending a single PHC facility in Pretoria, Tshwane during February 2018, the issue of interest (I): the screening for depressive features and its associated risk factors. Although there is no explicit comparison intervention of interest, the context (C) is that of a single PHC facility in Tshwane. The outcome of interest (O) would be the presence of depression and its associated risk factors.

This observational study therefore aimed to answer a narrow and specific question, relating to a specific group of patients attending a single PHC facility in a particular month. The authors implied that an additional objective would be to provide evidence to inform decision-making with respect to depression screening at PHC level. At this stage of the critical appraisal process, it remains to be seen if this focused and highly context-specific research question would be able to provide sufficient evidence to affect wider decision-making at other PHC facilities.

#### 2. Considering the introduction section in this article, identify three sentences or phrases that best reflect the authors’ starting point, from which the rationale for the research is further explained and/or elaborated (more than one correct answer possible). (3 marks)

Potential options include:

- Depression poses serious public health challenges globally. It affects the social, economic and clinical aspects of individuals, resulting in impaired physical health, poor health behaviours, increased financial costs and diminished role functioning.
- According to the South African Drug and Anxiety Group (SADAG), the depression cost of the country is about R218 billion because of presentism (attending work whilst unwell: R190bn) and absenteeism (unscheduled absence from work: R28bn).
- Primary healthcare is the initial area of contact between the patient and the healthcare system, and it could play a pivotal role in the management of depression. Depression can be detected and treated at a PHC setting where treatment is feasible, affordable and effective.
- Primary healthcare is the relevant area for screening, diagnosing and treating depression, but because of increasing workload at PHC settings, limited human resources and lack of screening tools, little time is available to screen for mental disorders and depression may go undiagnosed.
- Patients with depression are frequent users of medical services; therefore, PHC clinicians should actively seek to detect depressive disorders to prevent suicide and reduce healthcare costs.
- South Africa has implemented different strategies to improve mental healthcare within the country but there has not been a focus on routine screening at PHC facilities.
- A few studies have been conducted in South Africa focusing on validity of PHQ-9 as a screening tool for depression, but no recommendations were made for routine screening. A study by Anderson et al. in the Eastern Cape revealed that adult patients do not self-report depressive features despite going to PHC facilities frequently.

#### 3. Comment critically on the sample used in this study and any source of selection bias. (5 marks)

The study population comprised patients aged 18 years and older, who sought medical services at the clinic. This excludes patients younger than 18 years who are also at risk of depression. The authors did not justify this exclusion criterion; presumably, they were only focusing on adult patients who present to the PHC clinic for their usual care, but this is a limitation not mentioned. The other exclusion criteria (down-referrals from the psychiatric hospital and patients already diagnosed with depression at the clinic) make sense, as this study focused on screening for depression in patients not known to be depressive.

A sample size of 200 was calculated from the total head count of 7899, from April 2015 to April 2016. The authors did not specify the statistical method or formula chosen to calculate the sample size, nor the effect size or reference used to determine the detection rate of the screening tool (pre-study considerations of statistical power). It is also not clear how this head count of 7899 over a year relates to the head count for the single month of February 2018, as 200 patients were invited from presumably 1000 patients who presented to the facility and were not excluded based on the predefined exclusion criteria (a systematic random sampling technique was employed, where every 5th patient was selected). This means that the annual headcount should be closer to 12 000 (compared to 7899 for the period 2 years earlier), for adults with no existing mental health diagnosis (patients under 18 years excluded). This means that either the annual headcount from 2015 to 2016 may be inaccurate or that the systematic random sampling technique was not employed consistently.

Sources of selection bias could therefore include the following:

- This study includes a narrow focus (clinic based), rather than a broad focus (general population or community based) sample, when considering prevalence of undiagnosed depression in this setting. This brings the representativeness or generalisability of the study into question in the broader population. Admittedly, the focus of the study was screening for depression at PHC facilities.
- There may be a difference between those patients who are attending the PHC facility and those who are not presenting, as their risk factor profile may differ. For instance, the presence of chronic health conditions may predispose the patients attending the facility with a higher likelihood of associated depression, compared to patients who do not require regular health service access.
- As mentioned above, patients younger than 18 years were excluded, which limits the understanding of the overall prevalence of depression in this community of patients who make use of the healthcare services at this PHC facility.

#### 4. Critically appraise how well the authors describe the validity and administration of the study instrument identified for use in this study. (6 marks)

The authors stated that:

‘A modified PHQ-9 questionnaire was used as the screening tool. It was based on the two main questions from the DSM-IV [*Diagnostic and Statistical Manual IV*] criteria for major depression. … The first part of our questionnaire focused on the demographic data, potential risk factors and medical history. The second part of the questionnaire focused on depressive features for at least 2 weeks (PHQ-9), as well as functional health assessment.’

For a research tool or tool to be used in a study, it must be validated for the study setting. The authors state that the tool ‘has been proven to valid and reliable for use in primary care, … and resource-limited settings as well’. For this study, the tool is intended for screening adult patients not previously diagnosed with depression, to identify those that may have depressive symptoms and would require a more in-depth assessment according to the gold standard of diagnosing depression based on the DSM-IV criteria (the gold standard for the diagnosis of mental health disorders). They also state that the tool was modified and several references should be included, including reference 8, ‘Validity of the patient health questionnaire-9 to screen for depression in a high-human immunodeficiency virus (HIV) burden PHC clinic in Johannesburg, South Africa’, published in 2014. The reader is not guided on how the original PHQ-9 tool was modified for the local setting. Furthermore, it was not clear how the first part of the questionnaire (demographic data, potential risk factors and medical history) was developed to assist the authors with identifying risk factors in adult patients who screen positive for depressive symptoms. It appears that the first part of the questionnaire is separate from the previously validated second part of the questionnaire, which relates to the adapted PHQ-9. It is also not clear how the questionnaire collected data on ‘functional health assessment’. It would have been useful to provide the reader with access to the instrument as a supplementary file.

The authors state that the questionnaire was self-administered, but participants with poor English language ability were supported by a trained assistant or the principal investigator. It is not clear what percentage of participants received this support. It was also not clear how many participants had the necessary education level to complete the questionnaire independently (from the results section, we learn that around 12% of respondents had no schooling or only primary level schooling). Usually, the education level of the survey population should be considered when thinking about how easy it will be for respondents to interpret and answer a question. Ordinary and everyday language is preferred. Furthermore, it was stated that an interpreter was involved on an ad hoc basis, but again it is not clear how many participants required help with translation. The only statement was that the facility saw patients with a multi-lingual nature (the socio-demographic characteristics in Table 1 in the article being reviewed do not include home language, but state that around 75% of participants were South Africans and around 25% were non-South Africans). Questionnaires should be translated via a formal process of translating and back-translating to ensure that the original meaning for each item is retained, to ensure a consistent and reliable testing of the survey population. The authors argue in the limitations section that there was no need to translate the questionnaire as only two participants did not understand English.

Interestingly, the treating healthcare professionals were tasked with collecting the completed questionnaire in their consulting rooms. It is not clear whether the completed questionnaire was collected in a sealed envelope or ballot box to ensure anonymity. The possible under-reporting on some sensitive variables was mentioned in the limitations section. It would have been useful to consider a more anonymised and secure collection method, possibly via the trained research assistant.

#### 5. Critically appraise the reported response rate of 99.5% (3 marks)

A survey response rate of 50% or higher should be considered excellent in most circumstances. A high response rate is likely driven by high levels of motivation to complete the survey, or a strong personal relationship between the researcher or provider and the participant. Survey response rates in the 5% – 30% range are far more typical. Response rates are affected by the distribution method (face-to-face or via web or other interfaces), the simplicity of the tool (simple surveys with minimal questions are likely to attract better response rates than unwieldy and complex instruments) and the relationship between the respondent and the researcher/provider.

The authors reported a response rate of 99.5% (199 out of 200 invited participants completed the questionnaire). They did not report how many participants were excluded based on the exclusion criteria. Ideally, response rates should be reported fully, including details of participants who were unsuitable for the research or refused to take part. It may be that the prospective method of recruiting and screening participants as part of routine care could have benefited the response rate. The fact that the tool was self-administered in most respondents and that the completed tool was collected by the provider may also have benefited the response rate. However, the authors reported that the questionnaire took approximately 20–40 min to complete (which is fairly long for a self-administered tool) and it is not clear where the respondents were asked to complete the tool (in the waiting area waiting for their healthcare provider). It may be that the research assistant and/or principal investigator played a key role in ensuring a good response rate, especially if they were based at the facility during the month of data collection. The decision to contain the study to a single facility could also have contributed to the response rate, as well as the buy-in and support of the local healthcare team and facility management. In conclusion, a response rate of 99.5% is exemplary and warrants a discussion by the authors to clarify the possible reasons for such an outstanding result. Without such elaboration, the reader may be left wondering about how this response rate may have been achieved or if the denominator may have been incorrect.

#### 6. Critically appraise the authors’ decision to use a cut-off score of 5 to interpret the PHQ-9 score (3 marks)

A validated instrument has a standard guide to interpret the findings in a consistent manner across different study settings and populations. Regarding the interpretation of the study instrument, the authors used a cut-off score of 5 to calculate the screening outcome, which was considered as either positive for depressive features (score ≥ 5), or negative for depressive features (score < 5). Table 2 in the article being reviewed provides the PHQ-9 categories (from none to minimal to severe) and how they relate to the two screening outcome options (negative or positive). In the discussion section, the implication of using different cut-off scores was discussed. A higher cut-off score of 9 or 10 may have limited the number of ‘false positives’. The authors refer to the Wilson and Jungner principles of screening in the discussion section; however, for screening to be effective, the condition of interest should have a recognisable latent or early symptomatic stage. Screening for depression is a more complicated process, as there is no clear latent stage, and using a low score of ≥ 5 may help identify early depression. As mentioned in the discussion, using a cut-off score value which is too sensitive and results in ‘false positives’ may burden the patient with unnecessary exposure to antidepressants and add unnecessary pressure on the health system in a low-resource setting without adequate systems to respond to the patients who screen positive. Therefore, once a patient is screened positive, further clinical assessment and consideration of care options are warranted.

#### 7. ‘Employed participants had significantly lower odds (OR = 0.48) of screening positive (*p* = 0.033) for depressive features whilst the participants with significantly higher odds of being screened positive were those with co-morbidities (OR = 2.12; *p* = 0.029) and a history of stressful life events (OR = 3.21; *p* = 0.001)’. Define and explain what you understand by this statement? (5 marks)

Odds ratio is the odds of exposure in the diseased group divided by the odds of exposure in non-diseased group. It helps to identify risk factors related to a condition of interest (e.g. screening positive for depressive symptoms). It provides a method for comparing patients who already have a condition of interest (cases) with patients without this condition (controls).

In this study, the authors aimed to describe the risk factors associated with a positive screen for depressive features amongst patients. Based on the multivariate logistic regression model (Table 3 of the article being reviewed), the following OR relationships were found to be statistically significant: the OR of screening positive for depressive symptoms is lower in employed participants (unemployed participants are twice as likely to screen positive), whereas those with co-morbidities and a history of stressful life events were likely to screen positive two and three times, respectively.

Because only a sample of the population can be measured, confidence intervals (CI) (precision) give a range in which you think that the real answer lies with a given degree of certainty. For the OR in relation to employment status and screening positive for depressive symptoms, we can be 95% certain that the CI of 0.25–0.94 contains the true population parameter. For the OR in relation to the presence of co-morbid disease and screening positive for depressive symptoms, we can be 95% certain that the CI of 1.08–4.17 contains the true population parameter. For the OR in relation to the presence of a history of stressful life events and screening positive for depressive symptoms, we can be 95% certain that the CI of 1.64–6.28 contains the true population parameter. In general, the larger the sample size the smaller the CI, and vice versa. When the CI of a ratio crosses 1, that is, the range encompasses values showing increased and decreased risk, the statistical significance of the given ratio is weakened. It would have been useful had the authors reported the CI of the OR together with the *p*-value in the text description of the findings, as this will help the reader to interpret the OR related to risk factors for screening positive for depressive symptoms.

#### 8. Using the acronym READER to analyse this article’s applicability to your own context (take home message). (10 marks)

The READER format may be used to answer this question:

- Relevance to family medicine and primary care?
- Education – Does it challenge existing knowledge or thinking?
- Applicability – Are the results applicable to my practice?
- Discrimination – Is the study scientifically valid enough?
- Evaluation – Given the above, how would I score or evaluate the usefulness of this study to my practice?
- Reaction – What will I do with the study findings?

The answer may be a subjective response but should be one that demonstrates a reflection on the possible changes within the student’s practice within the South African public healthcare system. It is acceptable for the student to suggest how his or her practice might change, within other scenarios after graduation (e.g. general private practice). The reflection on whether all important outcomes were considered is therefore dependent on the reader’s own perspective (is there other information you would have liked to see?).

A model answer could be written from the perspective of the family physician employed in the district health system.

This cross-sectional study is relevant to the African primary care context, as screening, diagnosing and treating mental health conditions in an integrated manner represent core aspects of a high-quality, team-based PHC approach. The authors stated that they ‘envisage that the findings of our study may provide evidence to assist in decision-making with respect to screening for depression at PHC level’. Screening of depressive features at PHC facilities may have significant impact on improving detection rates, earlier diagnosis and improved outcomes. In terms of discrimination, the methodological concerns raised (sample size calculation, data collection process, response rate query and interpretation of findings based on the cut-off value) make any conclusions drawn questionable. Therefore, the most appropriate screening tool for use in South African PHC settings is not yet determined. The study may be discussed with the local and district management team and used as a basis for creating awareness regarding the undiagnosed and potentially unmet need of the mental health burden in PHCs. It is unlikely that this study will affect a change in policy direction, largely because of its study design limitations (cross-sectional study at a single PHC facility in one month), but it could help make the case for further research of a more robust design. The individual primary care provider and his or her team may be reminded about the potential risk factors for undiagnosed depressive symptoms, specifically in patients who present with chronic medical conditions, unemployment status and a history of recent stressful life events.

#### Further reading

- Mash B, Ogunbanjo GA. African primary care research: Quantitative analysis and presentation of results. Afr J Prim Health Care Fam Med. 2014;6(1):1–5. https://doi.org/10.4102/phcfm.v6i1.646
- Govender I, Mabuza LH, Ogunbanjo GA, Mash B. African primary care research: Performing surveys using questionnaires. Afr J Prim Health Care Fam Med. 2014;6(1):1–7. https://doi.org/10.4102/phcfm.v6i1.589
- Pather M. Evidence-based family medicine. In: Mash B, editor. Handbook of family medicine. 4th ed. Cape Town: Oxford University Press, 2017; p. 430–453.
- Naude C, Young T. How to search and critically appraise the literature. In: Goodyear-Smith F, Mash B, editors. How to do primary care research. 1st ed. Boca Raton, FL: CRC Press, 2019; p. 135–146.
- Stevens R. Statistics in primary care research. In: Goodyear-Smith F, Mash B, editors. How to do primary care research. 1st ed. Boca Raton, FL: CRC Press, 2019; p. 161–165.
- Ball L, Barnes K. How to conduct a survey in primary care. In: Goodyear-Smith F, Mash B, editors. How to do primary care research. 1st ed. Boca Raton, FL: CRC Press, 2019; p. 167–175.
- Joannabriggs.org. Critical appraisal tools – JBI [homepage on the Internet]. 2021 [cited 2021 Mar 16]. Available from: https://jbi.global/critical-appraisal-tools
- The Critical Appraisals Skills Programme (CASP). CASP checklists [homepage on the Internet]. 2021 [cited 2021 Mar 16]. Available from: https://casp-uk.net/casp-tools-checklists/
- Andersson LMC, Schierenbeck I, Strumpher J, et al. Help-seeking behaviour, barriers to care and experiences of care among persons with depression in Eastern Cape, South Africa. J Affect Disord. 2013;151(2):439–448. https://doi.org/10.1016/j.jad.2013.06.022

### Objectively structured clinical examination scenario

#### **Objective** of station

This station tests the candidate’s ability to provide chronic care to a patient with schizophrenia.

#### Type of station

Integrated consultation.

#### Role player

Young man or woman.

#### Instruction to candidate

You are the family physician overseeing the primary care clinic. The following patient, known with schizophrenia, is referred by the clinical nurse practitioner, as she is uncertain of the management plan.

Please consult the patient and manage accordingly.

As this is a chronic mental health consultation, a physical examination is not required.

## Instructions for the examiner

### Objectives

This station tests the candidate’s ability to provide chronic care to a patient with schizophrenia.

This is an integrated consultation station in which the candidate has 14 min.

**FIGURE 1:**
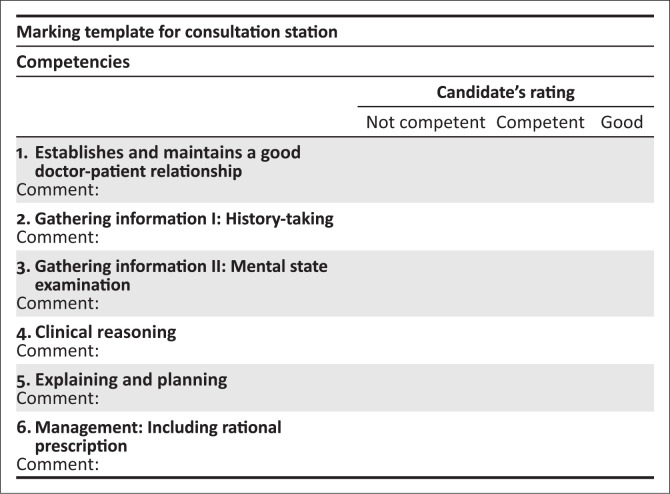
Marking template for consultation station.

- Familiarise yourself with the Assessor guidelines (Figure 1) which details the required responses expected from the candidate.
- No marks are allocated. In the mark sheet, tick off one of the three responses for each of the competencies listed. Make sure you are clear on what the criteria are for judging a candidates’ competence in each area.
- Provide the following information to the candidate when requested: (delete if not applicable).
- Please switch off your cell phone.
- Please do not prompt the student.
- Please ensure that the station remains tidy and is reset between candidates.
- This station is 15 min long. The candidate has 14 min, then you have 1 min between candidates to complete the mark sheet and prepare the station.

## Guidance for the examiner

**Working definition of competent performance:** the candidate ‘effectively completes the task’ within the allotted time, in a manner that ‘maintains patient safety’, even though the execution may not be efficient and well structured.

1. Establishes a good doctor–patient relationship
  The **competent candidate** acts within the ethical framework (respects autonomy, justice, non-maleficence and beneficence). In addition, the ‘good candidate’ displays empathy and compassion, acknowledging patient’s discomfort and the anxiety related to ongoing physical symptoms.
2. Gathering information I: History and examination findings
  The *competent candidate* gathers sufficient information to identify current medical issues ‘severe functional impairment due to tiredness and lack of motivation; using medication as prescribed; recent admission for substance-induced psychosis’ and identify any ongoing biopsychosocial risks. In addition, the ‘good candidate’ explores the patient’s experience, fears (‘fear of permanent disability due to persistent drowsiness and lack of motivation; employment prospects; fear of relapsing)’ and expectations, health seeking behaviour and identifies opportunities for health promotion ‘(maintenance plan for drug abstinence)’.
3. Gathering information II: Mental state examination
  The ‘competent candidate’ assesses the patient’s mental state ‘drowsy, blunted, low mood, cognitively intact but slow; good judgement and insight, including doing a risk assessment for harming self or other’. In addition, ‘the good candidate’ follows the recommended methodology for a mental state exam, displaying efficiency and confidence ‘assesses affect, mood, cognitive function, judgement and insight’.
4. Clinical judgement
  The ‘competent candidate’ uses available evidence to make the correct working diagnosis ‘overmedicated stable schizophrenia, with early signs of depression’. The ‘good candidate’ is able to make a comprehensive three stage assessment ‘as for “competent” + fear of disability; impact on occupational function; potential influence of contextual factors in relapse prevention’.
5. Explaining and planning
  The ‘competent candidate’ clearly explains the working diagnosis ‘no jargon; comprehensive; simple language’ and possible interventions. The ‘good candidate’ in addition provides a platform for the patient to engage as an equal partner in sharing information, and decision-making.
6. Management
  The competent candidate uses current evidence-based guidelines to develop a management plan (decreased dose of anti-psychotics and change to atypical anti-psychotic, e.g. risperidone; identifies risk of relapse on crystal methamphetamine, refers to psychotherapy or group support; and provides safety netting). In addition, the good candidate develops a comprehensive plan using the biopsychosocial approach (as for ‘competent’ + counsels’ patient on risk of relapse; chances of employment and helps with occupational health referral, mentions/refers to multidisciplinary team; and identifies need for structured follow-up plan).

## Examination findings and investigations

No physical examination findings – as per mental state examination.

### Role play – Instructions for actor

#### Appearance and behaviour

Young man.

#### Opening statement

‘Dr, the nurse asked me to come to you. She wants you to change my medication’

#### History

- Open responses: Freely tell the doctor:
  ■ You have had four admissions for psychosis, all induced by using crystal meth.
  ■ Your last admission was a month ago, and you were discharged 2 weeks ago – you haven’t touched any drugs since your admission, and want it to stay that way.
  ■ Your medication currently is haloperidol 5 mg three times a day.
- Closed responses: Only tell the doctor if asked:
  ■ Fears: (1) you are very worried that this sickness is going to lead to permanent disability – you have one child, 4 years old, and are unable to provide for her, (2) you want to stay off drugs, but live in a suburb with very easy access and surrounded by friends who are regular users.

#### **Social** history

- Single, living with parents and three younger siblings in a two-bedroom cottage in the township – you have electricity and running water. Your father is the breadwinner. You have never worked – dropped out of law school after first psychosis.
- Daughter lives with her mother – you are not involved.

#### If the doctor asks specific questions

- Mood: You feel ok, but worried all the time, and not motivated to do anything. You feel tired and want to sleep and eat constantly.
- Thoughts: You are fully in control of your thoughts. You have no delusions.
- Perception: No hallucinations of any kind.
- Insight: Understand that you have schizophrenia, a chronic incurable disease, worsened by drug use.
- Judgement: Recognise that you need help, as in the past you did not.

#### Further reading

- South African National Department of Health: Essential Drugs Programme. Schizophrenia. In: Hospital level (Adults) Standard Treatment Guidelines and Essential Medicines List. 4th ed. Republic of South Africa: National Department of Health; 2015:15.14–15.16.
- The psychiatric assessment. In: Swash M, Hutchinson R, editors. Hutchinson’s Clinical Methods. 19th ed. London: WB Saunders, 1989; p. 40–46
- Division of Clinical Pharmacology. Psycholeptics. In: South African Medicines Formulary. 9th ed. Cape Town: SAMA, 2010; p. 463–467

